# Nuanced Public Support for Rationing Treatments by Withdrawing and Withholding Due to Negative Reimbursement Decisions

**DOI:** 10.1007/s11673-025-10469-w

**Published:** 2025-09-10

**Authors:** L. Strand, L. Sandman, A-C. Nedlund, G. Tinghög

**Affiliations:** 1 Swedish National Centre for Priorities in Health, Department of Health, Medicine, and Caring Sciences, Linköping University, 581 83 Linköping, Sweden; 2 Department of Management and Engineering, Linköping University, 581 83 Linköping, Sweden

**Keywords:** Priority setting, Behavioural experiment, Health policy, Equivalence thesis, Resource allocation

## Abstract

When treatments are deemed not to be cost-effective and face non-reimbursement, policymakers in publicly funded healthcare systems may decide to ration treatments by withholding it from future patients. However, they must also address a critical question: should they also ration treatments by withdrawing it from patients already having access to the treatment, or is there an ethical difference between withdrawing and withholding treatments? To explore this question, we conducted a behavioural experiment (n=1404), examining public support for withdrawing and withholding treatments in reimbursement decisions across eleven different circumstances. Overall, public support for rationing by withdrawing and withholding was low, with no general perceived difference between withdrawing and withholding treatments. However, when we analysed the different circumstances separately, there were multiple circumstances where withholding was deemed ethically more problematic than withdrawing. Moreover, there was an overall preference for allowing individual assessments compared to ensuring that treatments are equally rationed between different healthcare providers. This result may indicate a preference for procedural fairness compared to outcome fairness. In addition, it was deemed more important to allow for individual assessments and to ensure equal rationing when withdrawing treatments compared to withholding. Overall, these findings reveal nuances in public preferences regarding withdrawing and withholding treatments, challenging the prevailing beliefs that withholding treatments is psychologically easier and ethically less problematic than withdrawing. They also challenge assertions of ethical equivalence between these two rationing approaches. If policymakers want to align their policies with public attitudes, our results suggest adopting a nuanced approach towards withdrawing and withholding treatments, recognizing that public support for ethical equivalence between withdrawing and withholding treatments varies depending on the circumstances.

**Supplementary Information:**

The online version contains supplementary material available at 10.1007/s11673-025-10469-w.

## Introduction

The imbalance between limitless healthcare needs and finite resources presents significant challenges for publicly funded healthcare systems. Not every medical treatment can be reimbursed, resulting in some treatments needing to be rationed, i.e., some patients are denied access to potentially beneficial treatments. In some cases, patients may access new promising treatments that have not yet undergone assessment for reimbursement eligibility. When such new treatment is later prioritized and reimbursed, both current and future patients can access treatment without ethical concerns arising. However, difficulties arise when medical treatments are not prioritized, and hence not reimbursed (see e.g., National Institute for Health and Care Excellence 2018). This situation leads to an ethical dilemma: should treatment be rationed by both *withholding* it from future patients and by *withdrawing* it from patients currently receiving treatment? This article presents a behavioural experiment that examines public support for withdrawing and withholding treatments in the context of reimbursement decisions, considering potentially morally relevant circumstances.

Traditionally, ethical differences between withdrawing and withholding treatments have been discussed in the context of rationing intensive care in intensive care units (ICUs). Previous research from this area suggests that both the public and professionals generally view withholding treatment as less problematic than withdrawing it when ICU resources are scarce and cannot meet the needs of all patients (Lin, Vitcov, and Cummings 2022). However, rationing at ICUs is a specific context which differs from rationing due to reimbursement decisions. Reimbursement decisions are made at a policy level far from the affected patient groups and healthcare professionals. In addition, they involve comparisons between the needs of current and future patients, either within a single patient population or across multiple populations. Cost-effectiveness may also be one relevant consideration along with severity, needs, rarity etc. In comparison, ICU decisions are made at the bedside level, which typically focus on the immediate needs of current patients within the same population. It is also difficult to assess cost-effectiveness or consider the needs of patient groups outside the ICU. Moreover, in our study, the ethical concern lies in differences between withdrawing and withholding for treatments deemed medically effective but not cost-effective. In ICU-related studies, the focus is often on medical efficacy when considering ethical differences between withdrawing and withholding (Levin and Sprung 2005; Ursin 2019; Vincent 2005). Furthermore, the nature of treatments differs. We focus on reimbursement decisions for continuous treatments for less acute conditions whereas ICU treatments are more short-term interventions for acute conditions. Lastly, the cause of scarcity differs. In reimbursement decisions, the scarce resource is typically money to finance drugs, where money can possibly be perceived as a more flexible resource, whereas in ICU decisions, resources such as beds and ventilators, are absolutely limited and, to a greater extent, fixed. Consequently, results from the ICU context may not be generalizable to reimbursement decisions.

Policy decisions not to reimburse treatment due to lack of cost-effectiveness typically lead to rationing by withholding future treatments. A contentious issue arises regarding whether treatments should also be rationed by withdrawing it from patients already having access to such treatments and whether such a policy would be accepted (Sandman and Liliemark 2018; Wester et al. 2021). Currently, there appears to be a widespread practice where treatments are not withdrawn after a negative reimbursement decision (see e.g., National Institute for Health and Care Excellence 2018). A study by van de Wetering, van Exel, and Brouwer (2017) asked participants to compare treatments currently in use and reimbursed over similarly effective treatments that are not yet in use or reimbursed. Their findings aligned with results from the ICU context, showing a preference for withholding over withdrawing. However, the study only implicitly examined differences between withdrawing and withholding treatments and not explicitly. Thus, it remains unclear whether there is a preferential difference between rationing by withdrawal and withholding in reimbursement decisions.

A previous interview study revealed that circumstances play a significant role in whether rationing by withdrawing and withholding in reimbursement decisions are viewed as ethically equivalent (Strand et al. 2022). For instance, participants emphasized that medically ineffective treatments should be rationed by both treatment withdrawal and withholding. However, withdrawing medically effective but non-cost-effective treatments was generally voiced as unacceptable, as it worsens the patient’s position and conflicts with fundamental human values and needs. While participants acknowledged no ethical difference between withdrawing and withholding medically ineffective treatments, differences emerged when cost-effectiveness was considered. Certain circumstances, such as a prior agreement between physician and patient about potential future withdrawal, were expressed as justifying the withdrawal of non-cost-effective treatments. Participants also emphasized that if non-cost-effective treatments are rationed, all clinics should follow the same procedures to ensure consistency and equality in decisions regarding withdrawal or withholding. Furthermore, participants highlighted the importance of national-level guidelines to support physicians in managing cases where treatments are not reimbursed due to a lack of cost-effectiveness and must be rationed by either treatment withdrawal or withholding (Strand et al. 2022).

In a subsequent study, we expanded on the findings of Strand et al. (2022) by conducting a behavioural experiment. Participants were assigned to one of three conditions: a withdrawing condition at the policy level, a withholding condition at the policy level, or a withdrawing condition at the bedside level. The experiment revealed that withdrawing treatments due to a lack of cost-effectiveness was deemed more problematic than withholding them. However, withdrawing treatments was considered less problematic at the bedside level than at the policy level (Strand et al. 2024). While these results indicate a preference for rationing by withholding over withdrawing—contrary to Strand et al. (2022)—the distinction between the policy- and bedside levels aligns with Strand et al. (2022), underscoring the significant role of contextual factors in shaping preferences for withdrawing and withholding treatments. In this article, we build on the findings of Strand et al. (2022) by conducting a behavioural experiment to examine public support for rationing by withdrawing and withholding treatments within the context of reimbursement decisions. Additionally, we explore how different possibly morally relevant circumstances shape support for rationing by withdrawing and withholding treatments.

## Methods

### Participants and Procedure

The experiment was programmed in Qualtrics, and data was collected during March 2023. We used the online research platform Prolific (Palan and Schitter 2018) to recruit a diverse U.K.-based sample of 1404 adults from their participant pool. Participants received a £2 reimbursement for participation. The average age of the sample was 39.90 (SD=13.13) and 58.62 per cent were female. A total of 55.84 per cent had completed higher education, while 25.07 per cent had completed further education. Additionally, 87.25 per cent of the participants successfully passed an attention check designed to assess data quality. The data, materials, and analysis codes can be accessed via this repository https://osf.io/y82qv/.

### Study Design

Participants were asked to indicate how much they agreed with eleven statements about rationing treatments on a seven-point Likert scale ranging from 1 = “completely disagree” to 7 = “completely agree.” Table 1 shows all statements. All statements were inspired by views expressed by physicians and patient representatives in our previous interview study, as described in the introduction (Strand, et al. 2022). This study builds on those qualitatively generated statements. We rephrased the statements whenever necessary to make them suitable for experimental testing. The order of the statements was randomized.

**Table 1:** Support with statements on rationing treatments by withdrawing and withholding

Statement		Withdraw (mean, SD)	Withhold (mean, SD)	*t*(df)	*p*.value
1	It is acceptable to [withdraw] (withhold) a treatment which is not medically effective.	6.04 (1.49)	5.84 (1.57)	−2.40 (1400)	0.017
2	It is acceptable to [withdraw] (withhold) a treatment which is medically effective but not cost-effective.	2.68 (1.46)	2.71 (1.52)	0.41 (1401)	0.68
3	A treatment that has proven to be effective for a patient participating in a clinical study, should be [withdrawn] (withheld) after the trial has ended as the cost-effectiveness of the medicine is still uncertain.	3.16 (1.60)	2.82 (1.48)	−4.19 (1390)	<0.001
4	If the physician has a previous agreement with the patient to [withdraw] (withhold) non-cost-effective treatments, then it is acceptable to [withdraw] (withhold) a medically effective treatment that is proven to be not cost-effective	4.38 (1.67)	3.99 (1.74)	−4.30 (1401)	<0.001
5	It is ethically acceptable to [withdraw] (withhold) a treatment when it is not cost-effective.	2.62 (1.46)	2.63 (1.52)	0.01 (1401)	0.99
6	It is important that different healthcare providers [withdraw] (withhold) treatments that are not cost-effective equally.*	3.84 (1.67)	3.56 (1.78)	−3.07 (1399)	0.002
7	It is important to allow individual assessments to affect whether to [withdraw] (withhold) treatments which are medically effective but not cost-effective.*	5.17 (1.51)	4.89 (1.63)	−3.26 (1397)	0.001
8	It is important to [withdraw] (withhold) treatments which are not cost-effective for all patients to uphold patient equality.	3.40 (1.59)	3.33 (1.71)	−0.77 (1397)	0.44
9	[Withdrawing] (withholding) a medically effective but not cost-effective treatment violates the human dignity of the patient.*	5.34 (1.44)	5.23 (1.55)	−1.41 (1397)	0.16
10	It is easier to [withdraw] (withhold) a medically effective, but not cost-effective, treatment if there are alternative treatments which the patient can get instead.	5.50 (1.47)	5.26 (1.68)	−3.03 (1398)	0.002
11	It is psychologically easy to [withdraw] (withhold) a treatment when it is not cost-effective.	2.29 (1.47)	2.36 (1.49)	0.79 (1402)	0.43

Note: The difference between withdrawing and withholding condition is highlighted in [brackets] and (parentheses). Participants answered on a seven-point Likert scale ranging from 1 = completely disagree to 7 = completely agree. A higher score implies greater support for rationing. *Item was reverse coded when averaging the statements and should be interpretated as a higher scoring indicating less support for rationing.

Participants were randomly assigned to either a withdrawing condition or a withholding condition. All aspects of the experiment were identical between the two conditions, except that participants in the withdrawing condition encountered statements framed as rationing by withdrawing, while participants in the withholding condition encountered equivalent statements framed as withholding treatment. For example, a statement in the withdrawing condition might read “It is acceptable to *withdraw* a medicine which is medically effective but not cost-effective” while the equivalent statement in the withholding condition would be phrased as “It is acceptable to *withhold* a medicine which is medically effective but not cost-effective.”

The data for this study was collected in the same survey as Strand et al. (2024). Thus, before responding to the eleven statement questions, participants answered a vignette question relating to withdrawing or withholding treatments. The vignette described a case in which patients with a serious medical condition required treatment. However, the treatment had been deemed non-cost-effective, meaning that resources spent on it could be better used elsewhere in the healthcare system. Withdrawing or withholding the treatment would result in patients not receiving potentially beneficial treatment, while providing the treatment would necessitate diverting resources from other areas of the healthcare system. Depending on the experiment condition, participants were then asked to express the acceptability of i) a policy to withdraw treatment, ii) a policy to withhold treatment, or iii) a bedside decision to withdraw treatment. Thus, before responding to this study’s eleven statements, participants were provided with detailed information about the specific context of rationing treatments due to reimbursement decisions. Throughout the current study and Strand et al. (2024), participants were consistently assigned to either the withdrawing or withholding conditions. The vignette is presented in Strand et al. (2024), and all study instructions are available in Online Supplementary Materials.

There were no statistically significant differences in demographics between the withdrawing and withholding conditions (see Table S1 in Online Supplementary Materials), indicating that randomization was successful. Consequently, any statistically significant differences in the experiment should not be attributed to demographic differences between the conditions.

### Analysis

We conducted *t*-test analysis for all individual statements to test whether there was a difference between the withdrawing and the withholding condition for each of the eleven statements. In addition, we calculated the average support for the eleven rationing statements, where a higher average score indicated a higher support for treatment rationing. Thereafter, we tested whether there were any general differences between withdrawing and withholding with a *t*-test. We also tested the robustness of the results by conducting ordinary linear regressions controlling for demographics and failed attention check. As some participants had previously been in a policy level condition compared to a bedside level condition (see Strand et al., 2024), we included a control variable for this previous manipulation.

### Ethics Approval

The study was not subject to ethics approval following Swedish research ethics legislation. This study was performed in line with the principles of the Declaration of Helsinki. The authors did not collect any sensitive information or any data which can be used to identify participants. There was no anticipated harms or discomforts associated with participation. Participants were reimbursed £2 (£12/h) for the approximately ten minutes it took to complete the survey.

## Results

Table 1 presents the average support for each of the eleven statements regarding rationing treatments through withdrawing and withholding in reimbursement decisions. Across conditions, the statements receiving the highest levels of support were those related to rationing due to lack of medical efficacy (Statement 1), rationing when alternative treatments are available (Statement 10), the notion that rationing due to cost-effectiveness violates human dignity (Statement 9), and the idea that rationing should allow for individual assessments (Statement 7). Conversely, the statements receiving the lowest levels of support included those related to rationing due to a lack of cost-effectiveness (Statement 2), and perceptions that rationing treatment is psychologically easy (Statement 11) or ethically acceptable (Statement 5).

On average, participants in the withdrawing condition expressed a support level of 3.61 points (SD=0.67) for rationing treatments, while those in the withholding condition showed a slightly lower average support of 3.57 points (SD=0.72). This difference was not statistically significant (*t*(1397)=1.18, *p*=0.24). The result remained similar when controlling for demographics, failed attention check, and previous manipulations (See Table S2 in Online Supplementary Materials). Thus, there was no overall difference between withdrawing and withholding treatments.^1^

### The Individual Statements

We compared the average support levels for each statement between the withdrawing and withholding condition to determine whether specific circumstances influenced the support for the two types of rationing. As shown in Table 1, a statistically significant difference was observed between withdrawing and withholding conditions for six out of the eleven statements. Notably, withdrawing was considered more problematic than withholding for statements: prioritizing equal withdrawal over equal withholding (Statement 6) and advocating for individual assessments (Statement 7). Conversely, withholding was viewed as more problematic than withdrawing in circumstances involving medical efficacy (Statement 1), withholding treatment post-clinical trial (Statement 3), previous agreements regarding rationing (Statement 4), or availability of alternative treatments (Statement 10). The analysis of these six individual statements shows that participants judged the appropriateness of the statements differently depending on whether they were framed as withholding or withdrawing. Furthermore, no statistically significant difference was observed between withdrawing and withholding when considering rationing due to a lack of cost-effectiveness (Statement 2), the ethical acceptability of treatment rationing (Statement 5), rationing treatments to uphold patient equality (Statement 8), that rationing violates the patient’s human dignity (Statement 9), or the psychological difficulty of treatment rationing (Statement 11). Thus, the analysis of individual statements revealed no consistent preferential difference for the acceptability of rationing treatments by withdrawing or withholding in the context of reimbursement decisions.

## Discussion

This study investigated public support for withdrawing and withholding treatments in reimbursement decisions across eleven different circumstances. Overall, our results revealed no general pattern of differences between participants who were faced with statements where rationing was framed in terms of withdrawing treatment compared to participants faced with statements where rationing was framed in terms of withholding treatment. When analysing the circumstances separately, there were four circumstances where withholding was viewed as more problematic than withdrawal; two circumstances where withdrawing was viewed as more problematic than withholding; and five circumstances where there was no statistically significant difference in acceptance of rationing between withdrawal and withholding conditions. These findings highlight the circumstantial complexity of decisions involving withdrawing and withholding treatments, underscoring the importance of nuanced approaches that integrate medical, ethical, and emotional considerations.

### A Nuanced (Non)-Difference between Withdrawing and Withholding

The analysis of individual statements demonstrated that different circumstances shape public support for withdrawing and withholding in varying ways. In four out of eleven circumstances**,** withholding was considered *more* problematic than withdrawing. A possible explanation is that initiating treatment allows for testing its medical efficacy, an option unavailable when treatment is withheld entirely. Consequently, starting treatment and later withdrawing it may be viewed as preferable to withholding treatment. This reasoning aligns with arguments from previous normative analyses (Vincent 2005) and interviews (Strand et al. 2022) for why withdrawing may be preferred to withholding treatments. Additionally, the potential presence of medical maximizers (see Scherer et al. 2016; Tinghög and Strand 2022) may explain why withdrawing was preferred to withholding, as such people are more likely to advocate for treatment benefits even in seemingly futile cases (Scherer et al. 2020).

Conversely, withdrawing was perceived as more problematic than withholding in two circumstances: the importance of healthcare providers rationing treatments equally (Statement 6) and allowing for individual assessments when rationing treatments (Statement 7). Psychological fallacies such as withdrawal-aversion (Wilkinson, Butcherine, and Savulescu 2019) and status quo bias (Ritov and Baron 1992), have often been proposed as explanations for why withdrawing might be viewed as more problematic than withholding. These psychological fallacies would predict that withholding should, to some extent, consistently be viewed as more acceptable than withdrawing by the public. However, our findings challenge this notion, as withholding was preferred in four out of the eleven examined circumstances, and no statistically significant difference between withdrawing and withholding was observed in five circumstances.

The absence of statistically significant differences in five circumstances suggests a potential view of ethical equivalence between withdrawing and withholding treatments in these cases. This result diverges from previous quantitative empirical research in the setting of reimbursement decisions (Strand et al. 2024; van de Wetering et al. 2017) or from the ICU-setting, where withholding is preferred to withdrawing (Lin et al. 2022). However, it aligns with qualitative research (Spranzi, Morinet, and Foureur 2025; Strand et al. 2022) and with normative analyses proposing ethical equivalence between withdrawing and withholding (Sandman and Liliemark 2018; Wilkinson et al. 2019) from research regarding both reimbursement and ICU decisions. Taken together, the analysis of the eleven circumstances points to nuances rather than a consistent patterns in public support for withdrawing versus withholding treatments due to negative reimbursement decisions.

Our results did not align with Strand et al. (2024), a related study using the same sample, as we found no general difference in acceptance levels between withdrawing and withholding treatments. Additionally, in four circumstances, withholding was deemed less acceptable than withdrawing. Strand et al. (2024) examines a specific and detailed case of withdrawing and withholding treatments, explicitly informing participants about opportunity costs, the meaning of cost-effectiveness, and the concrete rationing decision faced by a public health agency. In contrast, the current study explores preferences using brief, general statements about rationing treatment through withdrawal and withholding, covering multiple circumstances and factors not included in Strand et al. (2024). Given these differences in study design (and research questions), results may diverge. However, the two studies’ approaches may complement each other—one providing specificity while the other captures more general opinions. Overall, the results suggest that when forced to state the acceptability of rationing within a more specific and detailed situation, withdrawing is perceived as less acceptable than withholding. However, when evaluating preferences for withdrawing versus withholding across a range of brief generic statements, no clear general preference for either withdrawing or withholding emerges.

### A Preference for Individual Assessments over Equal Access to Care and Resource Efficiency

A noteworthy finding from this study is participants’ support for allowing individual assessments to be made (Statement 7) paired with some support for assuring that treatments are rationed equally (Statement 6) when a treatment is deemed to lack cost-effectiveness. Allowing for individual assessments indicates an acceptance of procedures that result in unequal access to care and health inequalities, as it leads to some patients getting access to treatments while other similar patients do not. Overall, these results suggest a preference for procedural fairness (Statement 7, making individual assessments) over outcome fairness or equal outcomes (Statement 6, healthcare providers rationing treatments equally). More specifically, the results indicate a preference for a specific procedural fairness where reimbursement decisions based on cost-effectiveness, given considerations of e.g., needs and severity, can be reassessed on a case-by-case basis for individual patients. There are three implications for allowing individual assessments on a case-by-case basis.

First, the “case-by-case re-assessment” procedure might indicate an awareness of the fact that decisions at group level will have outliers, if there is heterogeneity in how the patient population respond to treatment or the severity of their conditions. By allowing individual assessment, those patient outliers, where treatment would be cost-effective given severity (and other potential factors), are given a second chance.

Second, however, overruling a previously determined assessment of cost-effectiveness through subsequent individualized assessments can undermine the fairness and legitimacy of the reimbursement decision. The objective with reimbursement decisions based on cost-effectiveness *and* other values such as needs and severity, is to prioritize treatments to those with the highest needs while maintaining an efficient resource allocation. However, if physicians are allowed to re-assess considerations of cost-effectiveness and needs on a case-by-case basis, less overall health will be provided (as the treatment is not cost-effective) and less resources will be available for patients with higher needs (as the reimbursement decision already considered the patient group’s needs). Thus, implementing a “case-by-case” procedure defeats the purpose of the initial reimbursement decision.

Third, the “case-by-case re-assessment” procedure might indicate that respondents want to have the ability to change a decision when the outcome of applying a certain procedure was not in line with their view of an acceptable outcome. This might indicate a shift in preferences regarding the type of decision framework used to address fairness, be it procedural or outcome based; or an acceptance of a procedural framework insofar as it rhymes well with an accepted outcome. However, by re-assessing the first procedure in a second, individualized, procedure, there will likely be greater inequalities in access to care and less efficient resource allocations, impacting equality in access to care for other patients. Thus, allowing for individual assessments could be a case of inconsistent fairness preferences (see e.g., Bolton, Brandts, and Ockenfels 2005; Trautmann and Van De Kuilen 2016) and seems practically problematic to implement.

Interestingly, the two circumstances concerning individual assessments and equal rationing were the only occasions where withdrawing treatment was deemed more problematic than withholding. This may reflect the perceived importance of individual assessments in treatment withdrawal or the desire for consistency in healthcare rationing. However, as previously argued, such preference appears ethically inconsistent, as endorsing individual assessments may result in differences among healthcare providers in treatment withdrawal, thereby increasing inequalities in healthcare access. One possible explanation is that participants interpreted “rationing equally” as contingent on “relevant individual assessments” or similar conditions. Another explanation may be a prioritization of maximizing patient access to treatments (i.e., wanting to avoid healthcare rationing) without fully considering the associated ethical implications, for example, that other patients in the healthcare system, with potentially greater needs, will not get access to their treatments. Therefore, the stronger preference for making individual assessments and equal rationing when considering treatment withdrawal does not necessarily represent an ethical inconsistency but a preference which is not fully considered.

### Implications

Our results have several implications. Firstly, *if* policymakers aim to align their policies with public views, our results suggest they should adopt a more nuanced approach to withdrawing and withholding treatments than current policies allow (see e.g., National Institute for Health and Care Excellence 2018; Wester et al. 2021), accounting for varying circumstances. Unlike previous quantitative empirical investigations (see Lin et al. 2022; Strand et al. 2024; van de Wetering et al. 2017), the results from the analysis of individual statements suggest that public perceptions of the ethically acceptability of withdrawing and withholding treatments are highly context-dependent rather than inherently a case favouring one over the other. Secondly, the dichotomous distinction about the ethical equivalence or non-equivalence of withdrawing and withholding (see e.g., Sandman and Liliemark 2018; Ursin 2019; Wilkinson et al. 2019) may need to be reconsidered. Public support for equivalence seems more contingent on the specific circumstances than previously recognized. Thirdly, clinicians should recognize the challenges of withdrawing and withholding treatments based on lack of cost-effectiveness. Our findings indicate that the only broadly acceptable circumstance for rationing due to treatments not being deemed cost-effective was when there was a previous agreement regarding future withdrawal. Consequently, establishing such agreements before initiating treatment with unclear reimbursement status could enhance public support. While this approach has been criticized (Wester et al. 2021), our findings, alongside previous studies (Strand et al. 2022; Zhong et al. 2022) suggest such agreements may be publicly acceptable.

## Limitations

First, our results are mostly only applicable to the specific setting we have analysed, and are difficult to generalize to, for example, the ICU setting. Thus, any generalization of the results to other contexts must be made carefully. Second, the brief nature of our statements allowed room for interpretation. However, we aimed to remain as true as possible to the statements formulated in Strand et al. (2022) to quantitatively test findings from qualitative research. Participants also read a thorough vignette before answering the questions (see Strand et al. 2024) which contextualized the statements in the context of rationing due to reimbursement decisions. Any potential confusion regarding the statements likely affected experimental conditions equally. Third, the use of a Prolific sample may limit generalizability, as it is not nationally representative. However, recent studies have indicated that Prolific provides high-quality data (Stagnaro et al. 2024). Lastly, this study was conducted during a time of historically low support for the U.K. healthcare system (The Health Foundation 2022). This context may have influenced the low overall support for rationing, potentially limiting generalizability.

### Future Research

To mitigate ambiguities associated with brief statements, future research should develop more detailed vignettes (e.g., in the style of Strand et al. 2024) to explore how factors such as previous agreements, access through clinical trials, and opportunity cost neglect (see Maguire, Persson, and Tinghög 2023; Persson and Tinghög 2020) shape public support for withdrawing and withholding treatments. A comprehensive normative analysis of the results’ ethical implications would also provide valuable guidance for policymakers navigating the complex rationing dilemma of withdrawing and withholding treatments.

## Conclusions

This study examined public support for withdrawing and withholding treatments not deemed cost-effective across various potentially morally relevant circumstances. Our behavioural experiment showed nuanced public support for withdrawing and withholding treatments. There was no general preference for either withdrawing or withholding treatments, suggesting ethical equivalence between withdrawing and withholding. However, withholding was viewed as more problematic than withdrawing when considering medical efficacy, prior agreements about future withdrawal, access after clinical trials, and availability of alternative treatments. Moreover, the public were supportive of making individual assessments and less supportive of rationing equally, but both considerations were more important when withdrawing treatments. This result may indicate a preference for procedural fairness over outcome fairness, as well as being potentially ethically inconsistent. Withdrawing and withholding were seen as equally psychologically difficult, ethically unacceptable, and equally great violations of patients’ human dignity. Overall, public support for healthcare rationing by both withdrawing and withholding in the context of reimbursement decisions was low. The question of ethical equivalence between withdrawing and withholding treatments may lack practical relevance, as public attitudes appear somewhat nuanced and depend on specific circumstances. Policymakers may need to allow for a circumstantial clinical flexibility regarding this dilemma while exploring strategies, such as prior agreements on future withdrawal, to navigate the ethical and practical challenges of withdrawing treatments in reimbursement decisions.

## Supplementary Information

Below is the link to the electronic supplementary material.

Supplementary file1 (DOCX 49 KB)

## Data Availability

The study was pre-registered at https://osf.io/y82qv/; data, analysis codes, and supplementary materials (including experimental instructions and decision screens) can be accessed via this link.

## Footnotes

1 A Kolmogorov-Smirnov test showed a difference in distribution between the two variables (D(697, 707)=0.07, *p*=0.0495) (see Figure S1 in Online Supplementary Materials). However, upon visual analysis of the Figure S1, this seems to be a result of the test being sensitive, i.e., the distributions look fundamentally similar.
