# Supplementary material for: Nuanced Public Support for Rationing Treatments by Withdrawing and Withholding Due to Negative Reimbursement Decisions
Source: J Bioeth Inq. 2025 Sep 10;23(2):231–40. doi: 10.1007/s11673-025-10469-w (PMC13388724; doi:10.1007/s11673-025-10469-w)
Supplement: Supplementary file 1 — Supplementary file1 (DOCX 49 KB) [file 11673_2025_10469_MOESM1_ESM.docx]

Supplementary Materials – Nuanced Public Support for Rationing Treatments by Withdrawing and Withholding Due to Negative Reimbursement Decisions

Journal of Bioethical Inquiry.
**Strand L_1_***; Sandman, L_1_; Nedlund, A-C_1_; Tinghög, G_1,2_.

_1_Swedish National Centre for Priorities in Health, Department of Health, Medicine, and Caring Sciences, Linköping University, Sweden.
_2_Department of Management and Engineering, Linköping University, Sweden.
* correspondence [liam.strand@liu.se](mailto:liam.strand@liu.se)

# Supplementary Statistical Analysis

## Demographical Differences between the two conditions

As seen in Table S1**,** the average age was 39.90 (SD=13.13), 41.38% were male, the education level was high as 55.84% had completed higher education and 25.07% had completed further education. Moreover, 87.25% of the participants completed the attention check. A One-Way ANOVA show that mean age was not statistically different between the two experiment conditions (D(1, 1402), *F*=0.05, *p*=0.98). Furthermore, Chi-square tests show that there were no statistically significant differences for either gender (χ^2^(1, 1404)=0.76, *p*=0.38), education level (χ^2^(2, 1404)=2.59, *p*=0.27), or completed attention check (χ^2^(1, 1404)=0.07, *p*=0.79).

| Table S1: Descriptive Statistics | | | |
| --- | --- | --- | --- |
| Condition | Withdrawing condition | Withholding condition | All |
| Age, mean (SD) | 40.02 (13.23) | 39.77 (13.04) | 39.90 (13.13) |
| Male, % (n) | 42.6% (297) | 40.17% (284) | 41.38% (581) |
| <Secondary Education, % (n) | 18.08% (126) | 20.08% (142) | 19.01% (268) |
| Further Education, % (n) | 26.83% (187) | 23.33% (165) | 25.07% (352) |
| Higher Education, % (n) | 55.09% (384) | 56.58% (400) | 55.84% (784) |
| Completed Attention Check, % (n) | 86.94% (606) | 87.55% (619) | 87.25% (1225) |
| n | 697 | 707 | 1404 |

## Regression analysis

As seen in Table S2, we tested the robustness of the results by conducting regression analysis. The effect of withdrawing remained statistically insignificant (t=1.06, p=0.29) in Model 1 where we controlled for age, gender, and education level. Furthermore, as seen in Table S2, Model 2 tests the robustness by excluding participants who failed an attention check. Nonetheless, the effect of withdrawing remained statistically insignificant (t=1.07, p=0.28).

Notably, Table S2 shows in both models that a higher age was associated with a higher support for rationing treatments. Being male was in both models associated with a higher support for rationing treatment. Having completed further education was in both models associated with a lower support for rationing compared to have completed higher education. To at most have completed secondary education was in Model 2 associated with a lower support for rationing compared to a higher education. Participants had previously answered a question related to withdrawing and withholding at either the bedside or policy level. Model 3 displays the results of our main analysis when controlling for this potential order effect.

Withdrawing condition remained statistically insignificant (t=0.99, p=0.32). Interestingly, participants who had previously been in the policy level condition were on average 0.08 points less supportive of rationing at the bedside level. The result is consistent with the results from (Strand et al., 2024).

| **Table S2: Regression analysis on average support for rationing.** | | | | | | |  |  |  |
| --- | --- | --- | --- | --- | --- | --- | --- | --- | --- |
|  | Model 1 | | | Model 2 | | | Model 3 | | |
|  | Beta | SE | p-value | Beta | SE | p-value | Beta | SE | p-value |
| Withdrawing condition | 0.04 | 0.04 | 0.29 | 0.04 | 0.04 | 0.29 | 0.04 | 0.04 | 0.32 |
| Age | 0.00 | 0.00 | 0.009 | 0.06 | 0.00 | 0.002 | 0.00 | 0.00 | 0.009 |
| Male | 0.24 | 0.04 | <0.001 | 0.24 | 0.04 | <0.001 | 0.24 | 0.04 | <0.001 |
| <Secondary Education | -0.07 | 0.05 | 0.19 | -0.11 | 0.05 | 0.034 | -0.07 | 0.05 | 0.17 |
| Further Education | -0.09 | 0.04 | 0.04 | -0.11 | 0.05 | 0.020 | -0.09 | 0.04 | 0.044 |
| Policy Level |  |  |  |  |  |  | -0.08 | 0.04 | 0.022 |
| Intercept | 3.36 | 0.07 | <0.001 | 3.33 | 0.07 | <0.001 | 3.36 | 0.07 | <0.001 |
| n | 1404 | | | 1225 | | | 1404 | | |
| Note: All regressions are ordinary least square with robust standard errors. Acceptance for rationing was measured as the average of 11 statements measured on a scale from completely disagree (=1) to completely agree (=7). Higher education (>3 years) is the reference group for education. Policy level means that in participants had previously answered related to withdrawing/withholding at the policy level compared to the bedside level. Model 2 excludes participants who answered wrong on the attention check. Model 3 controls for the policy level order effect. | | | | | | | | | |

Figure S1 displays the average support for the rationing statements, ranging from 1 to 7. The there is a large overlap (the burgundy-coloured area) between withdrawing and withholding condition, with withholding (grey) having somewhat lower support. Overall, the two distributions look fairly similar.


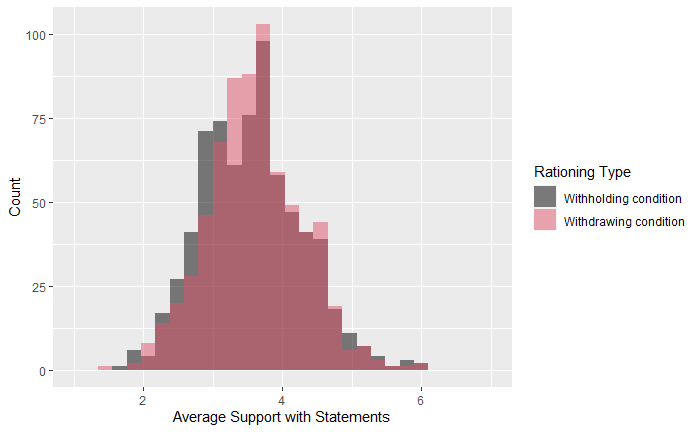


**Figure S1**. Distribution of the average support for rationing treatments.

# Transcript of Survey

*INSTRUCTIONS FOR ALL CONDITIONS*

Welcome!

If you agree to participate in this study, we will ask you several questions related to your attitudes towards prioritisation of limited health care resources in different circumstances.

Your answers will be treated so no unauthorised persons access them. All publication of this research will only report results on an aggregated level or completely anonymized examples which will not identify you.

Participation in the study should take about 10 minutes and you will receive a participation fee of £2 for completing the entire study.

Please note: During the survey, there will be an attention check to ensure that you are paying attention to the study instructions.

There are no foreseeable risks to your participation in this study. By clicking the button below, you indicate that you understand the information that was presented and that your participation is voluntary, and you may withdraw your consent and discontinue participation in the project at any time.

- I consent to participate in this study

Please enter your Prolific ID

________________________________________________________________

## [The withdrawing condition]

On the next screen, you will answer several questions about how the health care provides treatments, which in some cases are not deemed cost-effective. Withdrawing a treatment means that the treatment is withdrawn from patients who currently undergo treatment, i.e., they will no longer have access to it.

[NEW SCREEN]

Please indicate to what extent you agree with the following statements in a publicly funded health care system. Keep in mind that there are no right or wrong answers.

(Participants answered these questions on a 7-point Likert scale: 1=Strongly disagree, 2, 3, 4, 5, 6, 7=Strongly agree)

- It is acceptable to withdraw a treatment which is not medically effective.
- It is acceptable to withdraw a treatment which is medically effective but not cost-effective.
- It is easier for both the physician and the patient when withdrawing a medically effective, but not cost-effective, treatment if there are alternative treatments which the patient can get instead.
- It is important that different healthcare providers withdraw treatments that are not cost-effective equally.
- A treatment that has proven to be effective for a patient participating in a clinical trial, should be withdrawn after the trial has ended as the cost-effectiveness of the treatment is still uncertain.
- If the physician has a previous agreement with a patient to withdraw non-cost-effective treatments, then it is acceptable to withdraw a medically effective treatment that is proven to be not cost-effective.
- Withdrawing a medically effective but not cost-effective treatment violates the human dignity of the patient.
- It is important to allow individual assessments to affect whether to withdraw treatments which are medically effective but not cost-effective.
- It is important to withdraw treatments who are not cost-effective for all patients to uphold patient equality.
- It is psychologically easy to withdraw a treatment when it is not cost-effective.
- It is ethically acceptable to withdraw a treatment when it is not cost-effective.

[NEW SCREEN]

(Attention check)

In the previous question on how the health care provides treatments, what would happen with the patients’ treatment?

- It would be withdrawn (correct answer)
- It would be withheld
- It would be financed
- It would be reimbursed

## [The withholding condition]

On the next screen, you will answer several questions about how the health care provides treatments, which in is some cases are not cost-effective. Withholding a treatment means that the treatment is withheld from patients who currently seek treatments, i.e., they will not get access to it.

[NEW SCREEN]

Please indicate to what extent you agree with the following statements in a publicly funded health care system. Keep in mind that there are no right or wrong answers.

(Participants answered these questions on a 7-point Likert scale: 1=Strongly disagree, 2, 3, 4, 5, 6, 7=Strongly agree)

- It is acceptable to withhold a treatment which is not medically effective.
- It is acceptable to withhold a treatment which is medically effective but not cost-effective.
- It is easier to for both the physician and the patient when withholding a medically effective, but not cost-effective, treatment if there are alternative treatments which the patient can get instead.
- It is important that different healthcare providers withhold treatments that are not cost-effective equally.
- A treatment that has proven to be effective for a patient participating in a clinical trial, should be withheld after the trial has ended as the cost-effectiveness of the treatment is still uncertain.
- If the physician has a previous agreement with the patient to withhold non-cost-effective treatments, then it is acceptable to withhold a medically effective treatment that is proven to be not cost-effective.
- Withholding a medically effective but not cost-effective treatment violates the human dignity of the patient.
- It is important to allow individual assessments to affect whether to withhold treatments which are medically effective but not cost-effective.
- It is important to withhold treatments who are not cost-effective for all patients to uphold patient equality.
- It is psychologically easy to withhold a treatment when it is not cost-effective.
- It is ethically acceptable to withhold a treatment when it is not cost-effective.

[NEW SCREEN]

(Attention check)

In the previous question on how the health care provides treatments, what would happen with the patients’ treatment?

- It would be withdrawn
- It would be withheld (correct answer)
- It would be financed
- It would be reimbursed

## [Demographics]

*INSTRUCTIONS FOR ALL CONDITIONS*

[NEW SCREEN]

How old are you?

(The participants dragged a slider ranging from 18-100 with intervals of to indicate their age)

What’s your gender?

- Male
- Female

What’s your highest completed education?

- Primary education
- Secondary education (completed year 13)
- Further education (post-secondary and tertiary education)
- Higher education (at least Bachelor’s degree)

[NEW SCREEN]

We thank you for your time spent taking this survey.

Your response has been recorded.

# References

Strand, L., Sandman, L., Persson, E., Andersson, D., Nedlund, A.-C., & Tinghög, G. (2024). Withdrawing versus Withholding Treatments in Medical Reimbursement Decisions: A Study on Public Attitudes. *Medical Decision Making, 0*(0), 0272989X241258195. doi:10.1177/0272989x241258195
